# Polycystic Ovary Syndrome in Adolescence: Challenges in Diagnosis and Management

**DOI:** 10.1055/s-0042-1742292

**Published:** 2022-05-27

**Authors:** Mariana Enxuto Santos Manique, Ana Margarida Antunes Póvoa Ferreira

**Affiliations:** 1 Department of Ginecology and Obstetrics, Faculty of Medicine, Universidade do Porto, Porto, Portugal; 2 Department of Gynecology, Unit of Reproductive Medicine, Centro Hospitalar Universitário São João, Porto, Portugal; 3 Department of Gynecology, Obstetrics and Pediatrics, Faculty of Medicine, Universidade do Porto, Porto, Portugal; 4 Institute for Investigation and Innovation in Health, Universidade do Porto, Porto, Portugal

**Keywords:** polycystic ovary syndrome, adolescence, diagnosis, management, síndrome do ovário policístico, adolescência, diagnóstico, gestão

## Abstract

Diagnosing polycystic ovary syndrome (PCOS) during adolescence is challenging since normal pubertal development overlap typical features of this syndrome. The authors aim to summarize the existing evidence concerning PCOS in adolescence, particularly its diagnostic criteria and therapeutic options. A search throughout medical databases such as PubMed and MedScape was performed. Diagnostic criteria include irregular menstrual cycles according to time postmenarche and evidence of clinical hyperandrogenism and/or biochemical hyperandrogenism, provided other causes have been excluded. Polycystic ovarian morphology ought not to be used as a diagnostic criterion. Treatment should target manifestations and/or comorbidities, even in the absence of a definite diagnosis. Lifestyle interventions are the first-line treatment. Combined oral contraceptives, metformin or antiandrogens may also be considered as adjuvants. Screening for PCOS in adolescence is crucial as it allows an early intervention on the symptoms and comorbidities presented leading to better long-term reproductive and metabolic outcomes.

## Resumo

Diagnosticar a síndrome do ovário policístico (SOP) durante a adolescência é um desafio, uma vez que o desenvolvimento puberal normal se sobrepõe às características típicas desta síndrome. Os autores têm por objetivo resumir as evidências existentes sobre a SOP na adolescência, particularmente seus critérios diagnósticos e opções terapêuticas. Uma pesquisa em bases de dados médicas como PubMed e MedScape foi realizada. Os critérios de diagnóstico incluem ciclos menstruais irregulares de acordo com o tempo pós-menarca e evidência de hiperandrogenismo clínico e/ou hiperandrogenismo bioquímico, após exclusão de outras causas. A morfologia policística dos ovários não deve ser usada como um critério diagnóstico. O tratamento deve ser direcionado às manifestações e/ou comorbilidades, mesmo na ausência de um diagnóstico definitivo. As intervenções no estilo de vida são o tratamento de primeira linha. Contraceptivos orais combinados, metformina ou antiandrogênios também podem ser considerados como adjuvantes. O rastreamento da SOP na adolescência é fundamental, pois permite uma intervenção precoce ao nível dos sintomas e comorbilidades presentes levando a melhores resultados reprodutivos e metabólicos a longo prazo.

## Introduction

**Fig. 1:**
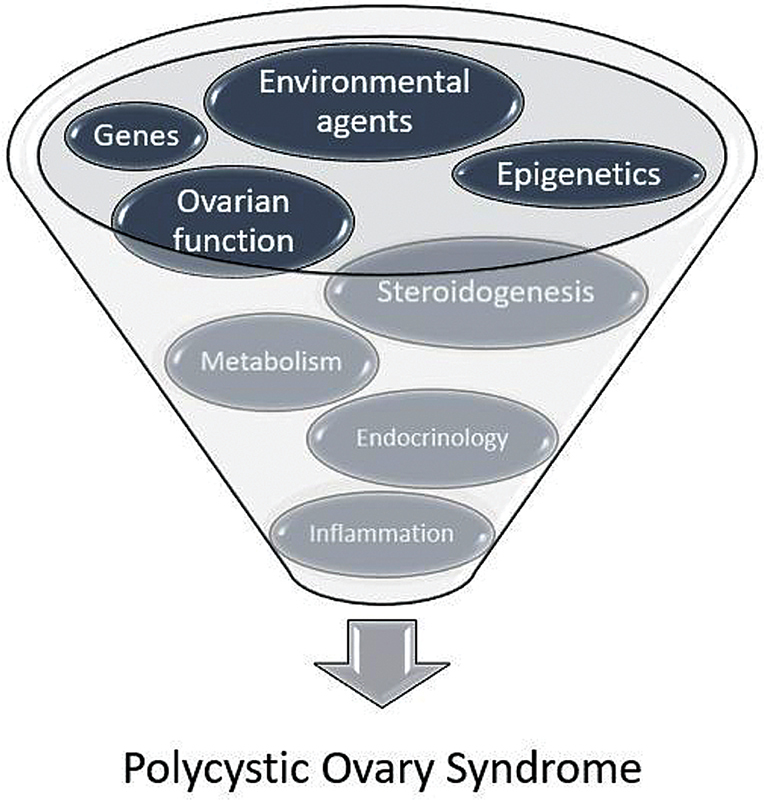
PCOS pathophysiology. Environmental agents comprise lifestyle factors such as food, exercise, stress, and endocrine-disrupting chemicals (estrogens, antiandrogens, bisphenol A, and other nutritional toxins). Endocrine factors include IR, hyperinsulinemia, nutrient excess, and ectopic fat storage. **Source:** Witchel et al. 2 and Ibáñez et al. 3

Polycystic ovary syndrome (PCOS) is the most frequent reproductive endocrine disorder affecting reproductive-aged women, being also the major cause of both chronic hyperandrogenic anovulation and infertility. Its estimated prevalence ranges from 6 to 15% depending on the populations studied and their ethnicity. Nevertheless, in adolescents, data concerning both incidence and prevalence is insufficient. 1 2 This syndrome has two main characteristic features: hyperandrogenism and ovulatory dysfunction. Clinical manifestations of hyperandrogenism include hirsutism and moderate to severe inflammatory acne. Ovulatory dysfunction may present as oligomenorrhoea or amenorrhea (primary or secondary). Among the main reproductive comorbidities are chronic anovulation, infertility, and pregnancy complications. Metabolic comorbidities include insulin resistance (IR), hyperinsulinemia, impaired glucose tolerance, type 2 diabetes mellitus (T2DM), gestational diabetes, hypertension, nonalcoholic fatty liver disease (NAFLD), dyslipidemias, metabolic syndrome, and an increased cardiovascular risk. Regarding psychological comorbidities, depression and anxiety are the most common, but eating disorders, negative body image, and sexual dysfunction may also be present. 2 Although the pathophysiology of PCOS remains unclear, it may result from multiple interactions ( Fig. 1 ) leading to heterogeneous manifestations among patients. 2 3 Even though it seems to mimic an autosomal dominant trait with variable penetrance, no single gene has yet been identified as responsible for all manifestations. 4

**Fig. 2:**
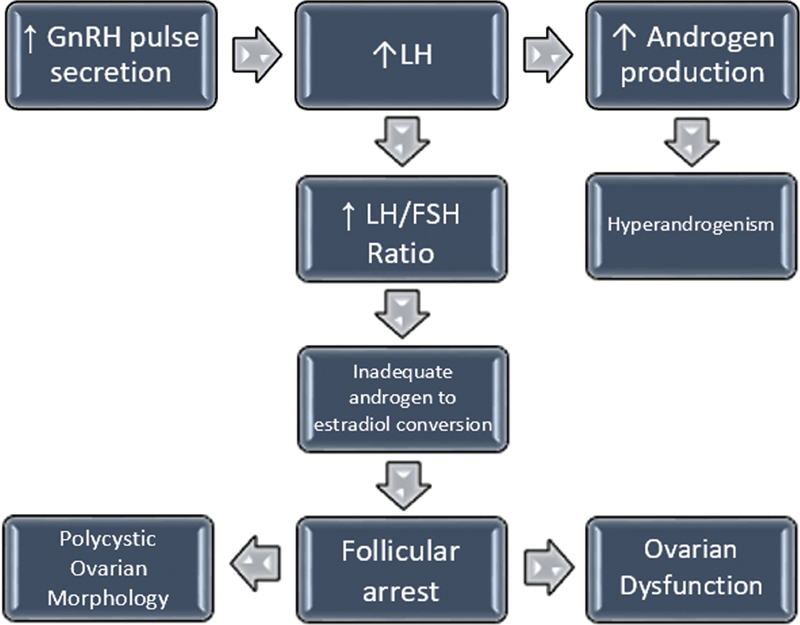
Hypothalamic-pituitary-ovarian axis dysfunction in PCOS. Abbreviations: FSH: follicle-stimulating hormone; GnRH: Gonadotropin-releasing hormone; LH: luteinizing hormone. **Source:** Witchel et al., 2 Ibáñez et al., 3 Fitzgerald et al. 4 and Trent and Gordon. 5

Under normal circumstances, there is a balance between growing and dormant follicles. In adolescent PCOS, several components of the hypothalamic-pituitary-ovarian axis are dysfunctional ( Fig. 2 ). 2 3 4 5 Insulin resistance with consequent hyperinsulinemia may play a role in its pathogenesis. It directly stimulates ovarian and adrenal androgen secretion and inhibits hepatic production of sex hormone binding-globulin (SHBG) with consequent increase of free testosterone concentrations. Insulin resistance together with parameters of oxidative stress also play a significant role in the development of metabolic comorbidities. Hyperandrogenemia per se might increase the production of free radicals, thereby disturbing redox balance toward the pro-oxidant state leading to early subtle clinical manifestations. 4 6

Potential risk factors for PCOS include low birthweight, *in uterus* exposure to androgens, postnatal rapid weight gain, adiposity rebounds at younger ages, early pubertal development with either premature menarche or pubarche, and adult weight gain with higher body mass index (BMI) values. 2 3 Interestingly, girls whose mothers have PCOS present with metabolic features even before the onset of hyperandrogenism. 7

## Diagnosis

Several diagnostic criteria have been proposed throughout the years. Notably, all require the exclusion of other potential causes of hyperandrogenism and ovulatory dysfunction. 8 In fact, conditions such as nonclassical congenital adrenal hyperplasia (NCCAH) – main differential diagnosis –, hypo or hyperthyroidism, pituitary disorders (hyperprolactinemia), hypothalamic amenorrhea, premature ovarian insufficiency, endogenous Cushing syndrome, and virilizing tumors, among others, must be ruled out. 9 10 11 Also, and since the main cause of amenorrhea in sexually active adolescents is pregnancy, the diagnostic workup should always include a pregnancy test. 10

The initial evaluation of a girl with signs and symptoms suggestive of PCOS begins with a precise medical history (including family history) and complete physical examination followed by appropriate laboratory assessment. 8 12 According to the most recent recommendations, this initial laboratory panel ought to include a pregnancy test, serum LH and FHS, as well as a complete blood count, comprehensive metabolic profile, and erythrocyte sedimentation rate. 9 It may also comprise thyroid function, prolactin, total testosterone, androstenedione, SHBG, dehydroepiandrosterone sulfate (DHEAS), and 17-hydroxyprogesterone concentration. Fasting glucose, glycated hemoglobin (HbA1c), and lipid concentrations are also typically requested. 12 13

The first criteria by the National Institute of Health (NIH) in 1990 established a PCOS diagnosis based on the presence of both clinical and/or biochemical hyperandrogenism and menstrual irregularity. 7 The Rotterdam criteria were developed in 2003 and further reformulated in 2004. 7 13 In 2006, the Androgen Excess Society (AES) came up with refined criteria. 7

Since there is a considerable convergence between normal pubertal milestones (such as acne, irregular menses, and polycystic ovaries) and PCOS phenotypes, experts considered the ahead criteria (NIH, Rotterdam and AES criteria) led to an overdiagnosis within this age group. 1 Therefore, several societies started including adolescents as a specific group within their guidelines for the diagnosis of PCOS. 7 10 13

The Amsterdam criteria, in 2012, was the first official consensus directed toward the specificities of adolescents, 13 14 followed by the Endocrine Society (ES) guidelines in 2013 13 and by an adolescent-specific expert consensus by the Pediatric Endocrine Society (PES) in 2015. 13 15

Also in 2015, the American Association of Clinical Endocrinologists (AACE), together with the American College of Endocrinology (ACE), the AES, and other PCOS societies created a practical guideline for evaluation and work-up of PCOS in this age group. They considered biochemical and/or clinical hyperandrogenism manifested as hirsutism and oligomenorrhoea for 2 to 3 years postmenarche as the basis of the diagnosis. Ultrasound should be excluded from the diagnostic criteria until the age of 17 years old. 14

Strong efforts have been made within recent years to overcome controversies from previous criteria resulting in three international consensuses for PCOS during adolescence (2015–2018). According to Rosenfield, 9 there are no discrepancies concerning the core diagnostic criteria ( Chart 1 ). Confusion emerges around their fulfilment. For instance, there is no agreement on the clinical manifestations of hyperandrogenemia or the time interval menstrual irregularities must persist until a definite diagnosis can be made. 9

**Chart 1:** Polycystic ovary syndrome diagnostic criteria in adolescence

Polycystic ovary syndrome diagnostic criteria in adolescence	
Hyperandrogenism *	- Evidence of clinical hyperandrogenism (moderate to severe hirsutism and/or severe acne) and/or - Evidence of biochemical hyperandrogenism
Ovulatory dysfunction *	- Irregular menstrual cycles according to time postmenarche: • 1 ^st^ year postmenarche: normal pubertal transition • From 1 to 3 years postmenarche: < 21 days or > 45 days • From 3 years postmenarche: < 21 or > 35 days or < 8 cycles per year • > 1 year postmenarche: > 90 days for any cycle • Primary amenorrhea: absence of menstruation by 15 years old or > 3 years postmenarche
Polycystic ovarian morphology	- Ought not to be used as a diagnostic criterion within this age group - Pelvic ultrasound should not be performed in adolescents < 8 years postmenarche.
Metabolic factors	- Metabolic criteria are not accepted - Metabolic factors should be considered as a warning sign to look for associated comorbidities

* Provided other causes are excluded.

**Source:** Rosenfield, 9 Akgül et al., 13 Peña et al. 16 and Witchel et al. 17

Therefore, all guidelines advocate for an evaluation 2 to 3 years after menarche whenever PCOS is a possible diagnosis. These girls should remain with a provisional diagnosis of “at risk” for PCOS. Definite diagnosis could be made afterwards in a retrospective way as long as the irregular menstrual cycles criteria persist according to time postmenarche. 12 17

Current recommendations reinforce the importance of establishing a balance: underdiagnosing this condition compromises early treatment and prevention of future complications; overdiagnosis has a great impact on both the physical and psychological health and on the well-being of the adolescent, with unnecessary exposure to side effects of certain medications. 2 4 As such, experts consider starting treatment targeted to the main manifestations and/or comorbidities presented by each patient, even in the absence of a definite diagnosis, which could be protruded to an older age as far as follow-up with careful monitoring is provided. In fact, recommendations do not require a definite diagnosis to effectively treat and manage young women presenting with typical features. 18

## Hyperandrogenism

DiVall et al. 10 defined hyperandrogenism as cutaneous evidence of excess androgens (excessive acne and/or hirsutism) or hyperandrogenemia (excess androgen levels in serum). It is the most common abnormality in PCOS, being present in between ∼ 60 and 80% of patients. 3 Although excessive ovarian androgen production represents most cases, increased adrenal androgen production can also occur. 3

Hirsutism, the primary and most reliable clinical marker for hyperandrogenemia, is the presence of excessive terminal hair growth with a male pattern. Due to the difficulties bellow presented in the biochemical evaluation of hyperandrogenemia (such as lack of a clear cuff point and the usage of male gender dosage callibration curves), its clinical evaluation is undoubtedly relevant, even with its inherent disadvantages. 6 Its main evaluation tool for diagnosis and follow-up is the modified Ferriman Gallwey (FG) score, whose cutoff depends both on the age and ethnicity of the girl. 6 16 In fact, it is expected that girls with Mediterranean, Hispanic or Middle Eastern origins develop a more severe hirsutism, whereas adolescents from East Asia tend to have milder forms. 19 The classical cutoff point of ≥ 8 is widely found in the literature to establish clinical hyperandrogenism. Nevertheless, and taking ethnical variations into consideration, Teede et al. 19 proposed a cutoff stratification. Accordingly, a score ≥ 8 should be restricted to those girls whose origins, as mentioned above, tend to developed a more severe hirsutism, whereas a score ≥ 6 should be applied in Caucasian and Afro descendant women, and ≥ 4 should be considered instead in Oriental/Asian girls. 6 19 In fact, and as defended by Soares-Jr et al., 6 the excess of body hair in women is a frequent problem at the clinic that interferes with their femininity and self-esteem and, therefore, should be appropriately diagnosed and managed. Nevertheless, using this score in females without complaints is controversial since a diagnosis of hirsutism may create a stigma and a strong emotional burden, especially within this age group, not to mention the exposure to unnecessary side effects of treatments. 6 In fact, milder forms of hirsutism may be considered normal within the Mediterranean, Middle Eastern or Latin American areas. 6 Furthermore, it does not take into consideration pubertal stages, has high interevaluator discrepancy, and is influenced by previous cosmetic treatments. 3 16 17 Finally, care must be taken to make a correct differential diagnosis between clinical hyperandrogenism and other forms of excessive hair growth, namely hypertrichosis and lanugo to avoid unnecessary treatments. 6

Hirsutism does not correlate directly with androgen circulating levels. 12 17 Therefore, whenever clinical hyperandrogenism is undetected, biochemical hyperandrogenism should be evaluated by high-quality assays, measuring total serum testosterone and SHBG. 3 16 Conversely, and since this conventional dosage calibration curve is the same used for the male gender, it is quite inaccurate for females, being unable to detect analytical evidence of hyperandrogenism in less severe forms of hirsutism. In fact, 30 to 50% of women with mild symptoms do not have corresponding elevated androgen levels. Another possible explanation for this finding relies on an exacerbated action of the androgens in effector organs (hair follicle) rather than on a significant elevation of its circulating levels. 6 Also, one must not forget that combined oral contraceptives affect SHBG and alter gonadotrophin-dependent androgen production. Thus, a reliable assessment of biochemical hyperandrogenism requires withdrawal of these therapeutic agents at least 3 months before measurement, providing another mean of contraception for sexually active adolescents. 18 19

Even though no clear cutoff points have been established, available guidelines recommend total testosterone concentrations > 55 ng/dl (1.91 nmol/l) for probable hyperandrogenism. 20 Ramezani Tehrani et al. 8 considered persistent elevation of serum total and/or free testosterone levels by >2 standard deviation (SD) above the mean of adult norms, determined by a reliable reference laboratory, as valid criterion.

Androstenedione and DHEAS provide limited additional information and are not recommended for the initial biochemical evaluation. They might be useful when testosterone levels are within the normal range for exclusion of other causes of hyperandrogenism, especially if an androgen-secreting tumor is suspected. 16

New highly specific and sensitive diagnostic tools are under investigation. One of these is anti-Müllerian hormone (AMH), which is produced by the granulosa cells of the ovaries, being involved both in the development and maturation of follicles. Anti-Müllerian hormone correlates with ovarian reserve, the number of developing follicles, and is a potential marker of ovarian aging. 17 Its concentration is frequently elevated in females with PCOS. Advantageously, it is not affected by the phase of the menstrual cycle. 4 21 However, there is still no cutoff validated for PCOS diagnosis in adolescent girls and its use is still controversial. 4 Khashchenko et al. 22 concluded that AMH as a sole marker for PCOS diagnosis in adolescents was insufficiently accurate. Accordingly, the consensus of opinion between pediatric endocrinologists and adolescent medicine experts considered that serum AMH concentration in adolescents should not be used to characterize polycystic ovarian morphology (PCOM) or to predict a diagnosis of PCOS. 18

Nevertheless, Dursun et al. 21 demonstrated that treatment with COC with or without metformin reduced AMH levels, independently of hyperandrogenism. Similarly, Asanidze et al. 23 showed AMH levels were reduced after treatment with combined oral contraceptives (COCs) or with COCs plus inositols. Hence, AMH might be a good marker for monitorization. 21 23

Finally, moderate to severe comedonal acne (that is, ≥ 10 facial lesions) or moderate to severe persistent inflammatory acne unresponsive to topical therapy is uncommon even within this age group and should prompt biochemical evaluation for hyperandrogenemia prior to medical treatment. 16 24

## Ovulatory Dysfunction

Whenever irregular menstrual cycles are present, a diagnosis of PCOS should be considered. 16 According to Peña et al., 16 irregular menstrual cycles are normal pubertal transition during the 1 ^st^ year postmenarche. After that, the following definitions of irregular menstrual cycles were reached by consensus, since there was insufficient data to formulate evidence-based ones: from 1 to 3 years postmenarche < 21 days or > 45 days; from 3 years postmenarche < 21 or > 35 days or < 8 cycles per year; and > 1 year postmenarche > 90 days for any cycle. 16 Primary amenorrhea is the absence of menstruation by the age of 15 years old or > 3 years post-thelarche 16 and, according to Javed et al., 25 it is associated with increased metabolic risk. Importantly, early menstrual patterns are predictive of the future ones and ovulation can occur even with irregular menstrual cycles. 12 In contrast, ovarian dysfunction may be present in females with regular menstrual cycles. 16

## Ovarian Morphology

In PCOS, ovarian cycles include the development of several primordial follicles but no dominant one is selected, leading to anovulation, atresia, and to PCOM. 26 Various parameters have been suggested to study ovarian morphology using ultrasonography (gold standard) in adults; however, no consensus concerning their diagnostic value in adolescents has yet been established. 27 Pelvic ultrasound might be used to better evaluate ovarian morphology and to exclude or investigate other possible uterine or ovarian abnormalities (functional cysts, ovarian masses, and endometrial alterations, among others). 16 However, it should not be used in females < 8 years postmenarche. 16 18 Polycystic ovarian morphology can be defined based on ovarian size and volume, stromal volume, and follicle size and number. The Rotterdam criterion defines ultrasonographic PCOM as a thickened capsule and enlarged ovary (> 10 cm ^3^ in volume), with multiple small cysts or ≥ 12 follicles that are 2 to 9 mm in diameter. 26 However, since ovaries reach their maximum volume and follicle count during puberty, this recommendation is not widely accepted and different sources suggest alternative dimensions specific for this age group. 26 27 The PES, for example, established a cutoff value of > 12 cm ^3^ rather than > 10 cm ^3^ for ovarian volume. 26 The AES guidelines, in turn, considered follicle number per ovary (FNPO) ≥ 25 could define PCOM. 26 Importantly, ultrasonographic findings are not specific for PCOS. 26 Furthermore, a high prevalence of girls has PCOM without an underlying pathology. Therefore, current recommendations advocate PCOM should not be considered as a diagnostic criterion of PCOS in this population. 16 18

## Metabolic Factors

Classically, PCOS is described as a primary ovarian disease. However, growing evidence shows neuroendocrine factors also play a role. In fact, in the presence of obesity, metabolic syndrome or IR, PCOS should be considered. 8 Polycystic ovary syndrome and metabolic syndrome have several common features such as obesity, IR, T2DM, hyperlipidemia, and hypertension. The prevalence of this syndrome is almost three times higher in women with PCOS. 1 12 However, data concerning its prevalence in adolescents with PCOS is discrepant. 15 First, the choice of diagnostic criteria for PCOS resulted in a great disparity in terms of the prevalence of metabolic syndrome ranging from 4.9 to 43.9%, being the highest when the PES criteria were used. This reinforces the importance of adolescent-specific definitions. 15 Second, there is still no consensus definition of metabolic syndrome in adolescents and published pediatric criteria are based on adult ones. 11 12 Nevertheless, metabolic syndrome is more prevalent among obese girls compared with lean adolescents when both are diagnosed with PCOS. During puberty, due to the increase in growth hormone (GH) levels, there is a physiological decrease in insulin sensitivity, which is also one of the reasons why PCOS may become clinically evident at this age. 1 3 12 However, IR is not necessarily present. Therefore, it should not be considered a diagnostic feature but rather a warning sign to look for associated comorbidities. 13 Even though there is data supporting a chronic low-grade inflammatory basis for PCOS, research concerning cytokine profiles within this syndrome turned out to be controversial. In fact, obesity itself is responsible for a proinflammatory state and, according to Barcellos et al., 28 independently of the presence of PCOS, circulating levels of interleukin 6 and high sensitive C-reactive protein are higher in obese females when compared with normal-weight ones. Therefore, the authors concluded that obesity, but not PCOS, affects circulating markers of low-grade inflammation in young women without major CV risk factors. 28 Also, higher levels of cystatin C (a proinflammatory marker related to low-grade inflammation) were found in girls with PCOS. This might be a promising early predictor of adverse cardiovascular outcomes, having a prognostic value and helping in risk stratification. 29 Similarly, progranulin levels are higher in these girls, being inversely correlated with HDL-C. Considering low HDL-C levels are a strong predictor of future cardiac events, progranulin might be used as a cardiovascular risk biomarker. 30

## Treatment

Usually, adult women with PCOS seek medical advice and/or treatment either due to menstrual dysfunction or to unsuccessful reproduction. However, the main concerns of adolescents are different and most often include irregular menstruation, acne, and hirsutism. 3 Treatment aims to improve both hormonal and metabolic status, quality of life, and long-term health status, preventing comorbid complications. 3 It should be started with education and lifestyle interventions. Classical pharmacological options include metformin, COCs, spironolactone, and topical drugs for hirsutism and acne ( Chart 2 ). 1 3 4 5 8 10 11 16 18 31 32 33 34

**Chart 2:** Classical treatment options (nonpharmacological and pharmacological) for polycystic ovary syndrome

**Lifestyle interventions (weight loss and physical activity)**	
Indications	- 1 ^st^ line nonpharmacological treatment - Recommended to all adolescents with polycystic ovary syndrome
Advantages	Weight loss: ✓ ↓ BMI ✓ ↓ FG score Physical Activity: ✓ Menstrual cycle regulation (↓ LH and ↓ AMH)
Disadvantages	✘ Suboptimal adherence ✘ High relapse rate
**Combined Oral Contraception (estrogen and progestin combinations)**	
Indications	- 1 ^st^ line pharmacological treatment - Menstrual irregularities and hirsutism - Contraception
Advantages	✓ Menstrual cycle regulation (↓ LH) ✓ ↓ Hyperandrogenemia ✓ ↓ Clinical manifestations of hyperandrogenism (seborrhea, acne, and hirsutism)
Disadvantages	✘ IR remains unchanged ✘ At least 6 to 9 months for measurable effects on hirsutism
**Antiandrogens (Spironolactone/Finasteride)**	
Indications	- Adjuvant to COC in severe hirsutism cases - COC contra-indication or not tolerated
Advantages	✓ ↓ FG score
Disadvantages	✘ Less effective for pre-existing hair ✘ Teratogenic
**Eflornithine (topical)**	
Indications	- Adjuvant to photoepilation in patients with laser-resistant facial hirsutism - Monotherapy whenever photoepilation is not recommended
Advantages	✓ ↓ Hirsutism
Disadvantages	✘ Relapse after discontinuation
**Metformin**	
Indications	- 2 ^nd^ line pharmacological treatment - Ineffective lifestyle interventions - COC contraindication or not tolerated
Advantages	✓ ↓ IR and hyperinsulinemia ✓ Menstrual cycle regulation ✓ ↓ Hyperandrogenemia ✓ ↓ Cardiovascular risk
Disadvantages	✘ Most symptoms relapse after discontinuation ✘ Side effects: gastrointestinal symptoms; lactic acidosis (extremely rare).

Abbreviations: BMI, body mass index; COC, combined oral contraceptive; FG, Ferriman Gallwey; IR, insulin resistance.

**Source:** Le et al., 1 Ibáñez et al., 3 Fitzgerald et al., 4 Trent and Gordon, 5 DiVall and Merjaneh, 10 Kostopoulou et al., 11 Peña et al., 16 Morin-Papunen, 18 Pal Singh Kochar et al., 31 Pkhaladze et al., 32 Abdolahian et al. 33 and Wong et al. 34

Lifestyles changes including weight loss and physical activity in obese or overweight adolescents remain the first-line treatment. According to Wong et al., 34 these are beneficial for weight control but have no effect on biochemical hirsutism. However, more recent results advocate lifestyle interventions may also improve clinical, hormonal, and metabolic features (such as IR), decreasing androgen levels and improving menstrual cycle patterns. 33 Nevertheless, there is a high degree of relapse and a suboptimal adherence. 1 32 34

As for therapeutic interventions, COCs with 20 to 35 µg of ethinylestradiol combined with a progestin are widely recommended as first-line pharmacological treatment. These agents target both menstrual dysfunction and clinical manifestations of hyperandrogenism, further providing hormonal contraception. In fact, COCs are considered the most effective treatment for hirsutism, with up to 70% improvement in unwanted hair growth. 5 10 Nevertheless, insulin sensitivity does not change. 5 10 It is essential to screen adolescents for possible contraindications prior to starting this treatment. 11 12

There are insufficient trials in adolescents with PCOS evaluating different COCs and the literature shows no significantly different hyperandrogenism outcomes either with an antiandrogenic progestin or a nonantiandrogenic one. Therefore, no COC is clearly superior to help decision-making. 3 Combined transdermal paths and vaginal rings might be adequate methods, but, once again, there is no superiority evidence. 5 Progestin-only contraception (such as intrauterine devices) may be an alternative first-line treatment given its low systemic side effects and high contraceptive effectiveness. However, it does not raise SHBG and may cause weight gain. 3

There are several other treatment options for hirsutism, including mechanical hair removal techniques, topical medications (eflornithine), light-based strategies, and antiandrogens, apart from the ahead mentioned COC. Light-based strategies (photoepilation by laser or electrolysis) should be considered as first-line treatment for localized hirsutism in PCOS. 3 5 11

Topical eflornithine is indicated as an adjuvant to photoepilation in patients with laser-resistant facial hirsutism or as monotherapy whenever these light-based strategies are not recommended. Even though promising, the duration of treatment is not clear yet. 3 5 11 Nevertheless, clinical results might take up to 8 weeks and a relapse is expected after discontinuation. 4

Antiandrogens are an adjuvant option to COC in severe cases of hirsutism to inhibit the development of new terminal hair. Spironolactone, the most commonly prescribed androgen receptor blocker, effectively reduces the FG score. However, this option should mainly be considered if hirsutism has not improved after 6 months of COC monotherapy and only if the patient clearly considers it a manifestation that makes her uncomfortable. Once again, limited evidence is available regarding its use in adolescents. 4 5 6 8 16

Finasteride, an 5α-redutase inhibitor, also plays an antiandrogen role, reducing dihydrotestosterone levels by between 50 and 60% with a consequent significant reduction in FG scores after 6 months of treatment. This agent should be considered mainly when prior therapy with COC and spironolactone were not effective for severe forms of hirsutism. Nevertheless, there is a paucity of literature concerning the use of antiandrogens within this population, namely in terms of safety and security. Furthermore, we must not forget that this therapy in adolescence could affect bone mineral density. 35

There are several pharmacological approaches available for acne, including topical retinoic acid and antibiotics (clindamycin, erythromycin, …) as well as oral antibiotics, hormonal therapy, and antiandrogens. However, retinoids are considered the most effective treatment. 5 8

Although insulin-sensitizing medications may have a positive impact on PCOS treatment, their use in adolescents is still controversial. Metformin reduces IR and, therefore, improves menstrual regularity and decreases hyperandrogenemia. Also, a decrease in cardiovascular risk is expected. However, conflicting results on weight loss remain among studies and effects on hirsutism are barely observed. 4 8 11 18 Nevertheless, Pal Singh Kochar et al., 31 for instance, advocated for its positive effects on hirsutism and, hence, this point remains controversial. Unfortunately, most symptoms relapse after discontinuation. Even though the severity, duration, and persistence of side effects can be highly variable, the dropout rate due to these is minimum and this medication could be considered safe in this age group. 31

Some authors do not recommend other insulin sensitizers, such as thiazolidinediones, for the treatment of PCOS in adolescents due to safety concerns, but this is also controversial. 1 11

Since adolescent girls with PCOS require long-term treatments, alternative therapies with less unwanted effects are under investigation ( Chart 3 ). 1 4 5 23 32 Even though data in adolescents is still very limited and further studies are needed, the following seem to be the most promising options.

**Chart 3:** New therapeutic options under investigations for polycystic ovary syndrome

**N-acetylcysteine**	- ↓ BMI, ↓ waist-to-hip ratio and ↓ waist circumference - ↑ Insulin sensitivity - ↓ Testosterone levels - Lesser side effects
**Inositols (myo-inositol and D-chiro-inositol)**	- ↓ Weight - Menstrual cycle regulation - ↑ Insulin sensitivity - ↓ Hirsutism - Virtual absence of side effects
**Vitamin D supplementation**	- ↓ Total testosterone - ↑ Insulin sensitivity - Menstrual cycle regulation
**Chromium supplementation**	- Menstrual cycle regulation - ↓ Free testosterone - ↓ IR (?) - No side effects
**Orlistat**	- ↓ Weight - ↓ Cardiovascular risk - Improved ovulation rates - Lesser side effects

Abbreviations: BMI, body mass index; IR, insulin resistance.

**Source:** Le et al., 1 Fitzgerald et al., 4 Trent and Gordon, 5 Amr and Abdel-Rahim 24 and Pkhaladze et al. 32

A recent study cited in an article by Trent et al. 5 published in 2020 compared N-acetylcysteine with metformin. It had very positive results in terms of BMI, waist-to-hip ratio, and waist circumference reductions. N-acetylcysteine also increased insulin sensitivity and decreased testosterone levels, thereby improving metabolic and hormonal profiles. Overall, this agent seems promising especially due to lesser side effects. 5

Inositols (myo-inositol and D-chiro-inositol) may also have a role in the treatment of adolescents with promising results regarding weight reduction, menstrual regularity, and improvement of insulin, androgen levels, and hirsutism. These may rise as new agents in this age group given their virtual absence of side effects. 1 Moreover, with the association of myo-inositol and COCs, positive results both in metabolic and androgen profiles are enhanced, and weight gain associated with COCs is balanced. 32 Also, the combination of myo-inositol with α-lipoic-acid reduces both IR and inflammation. 33 34 35 36

Vitamin D supplementation in PCOS may reduce total testosterone concentration but it has no effect on free testosterone nor on SHBG levels. A favorable effect on insulin sensitivity and on menstrual regularity has also been described. 4 Additionally, it seems to improve both acne and hirsutism. 5 However, controversies remain as some authors came up with opposing conclusions.

According to Amr et al., 24 chromium supplementation may reduce IR and improve both mean ovarian volume and total follicular count, with consequent better menstrual regularity in adolescents. It also decreased free testosterone levels, with no side effects being reported.

Finally, orlistat has been suggested for the long-term management of PCOS due to its potential to promote weight loss and improve metabolism, diminishing cardiovascular risk. It may also improve ovulation rates, with less side effects than metformin, for instance. 5

Unfortunately, due to insufficient interventional studies, it is not yet possible to determine which could be the most effective treatment and management approach for PCOS in adolescents. 18

## Conclusion

According to the most recent recommendations, the diagnostic criteria for PCOS during adolescence include irregular menstrual cycles according to time postmenarche and evidence of clinical hyperandrogenism and/or biochemical hyperandrogenism, provided other causes have been excluded. Polycystic ovarian morphology ought not to be used as a diagnostic criterion. Girls who have features of PCOS but do not meet diagnostic criteria should be labeled as “at risk” of PCOS and monitored carefully. Therefore, if the symptoms persist, a retrospective diagnosis could be made. Lifestyle interventions are the first-line treatment for most adolescents with PCOS. Pharmacological agents such as COCs, metformin or antiandrogens may also be considered as adjuvants.
